# Identification and Functional Characterization of Tissue-Specific Terpene Synthases in *Stevia rebaudiana*

**DOI:** 10.3390/ijms21228566

**Published:** 2020-11-13

**Authors:** Savitha Dhandapani, Mi Jung Kim, Hui Jun Chin, Sing Hui Leong, In-Cheol Jang

**Affiliations:** 1 Temasek Life Sciences Laboratory, 1 Research Link, National University of Singapore, Singapore 117604, Singapore; savitha@tll.org.sg (S.D.); mijung9204@gmail.com (M.J.K.); huijun@tll.org.sg (H.J.C.); singhui@tll.org.sg (S.H.L.); 2 Department of Biological Sciences, National University of Singapore, Singapore 117543, Singapore

**Keywords:** stevia, *Stevia rebaudiana*, terpenoids, terpene synthase, volatile organic compounds

## Abstract

In addition to the well-known diterpenoid steviol glycosides, *Stevia rebaudiana* (Stevia) produces many labdane-type diterpenoids and a wide range of mono- and sesquiterpenoids. However, biosynthesis of mono- and sesquiterpenoids in Stevia remains unknown. Here we analyzed the extracts of Stevia leaves, flowers, stems, and roots by Gas Chromatography–Mass Spectrometry and putatively identified a total of 69 volatile organic compounds, most of which were terpenoids with considerably varied quantities among the four tissues of Stevia. Using Stevia transcriptomes, we identified and functionally characterized five terpene synthases (TPSs) that produced major mono- and sesquiterpenoids in Stevia. Transcript levels of these Stevia *TPS*s and levels of corresponding terpenoids correlated well in Stevia tissues. Particularly, the root-specific SrTPS4 and SrTPS5 catalyzed the formation of γ-curcumene/zingiberene/β-sesquiphellandrene and α-longipinene/β-himachalene/himachalol as multifunctional sesqui-TPSs, respectively. Most of the *SrTPSs* were highly responsive to various environmental stresses in a tissue-specific manner. Taken together, our results provide new insights into how Stevia produces diverse terpenoids to confer differential responses to various environmental factors in each tissue.

## 1. Introduction

More than 1700 volatile organic compounds (VOCs) have been identified from 90 different plant families belonging to both angiosperms and gymnosperms [1,2]. In nature, VOCs play important roles in plants’ defenses against abiotic/biotic stresses, herbivores, and pathogens, as well as in pollinator attraction, and in plant–plant communication [3,4,5]. VOCs also possess immense economic importance as they are extensively used in cosmetics, food, and pharmaceutical industries. Based on their biosynthetic origins, plant VOCs can be divided into several classes: terpenoids, benzenoids/phenylpropanoids, fatty acid derivatives, and amino acid derivatives [2]. Terpenoids represent the largest and the most diverse class of plant metabolites. The majority of terpenoids are volatile and contribute significantly to the aroma of fruits, flowers, and essential oils [6].

In plants, terpenoids are produced by a highly diversified and large class of proteins called terpene synthases (TPSs) [7,8]. Biosynthesis of terpenoids are regulated by abiotic stress factors, such as temperature, light, drought, and salt, as well as by herbivores and microbes [9,10,11,12]. For instance, emission of α-pinene, sabinene, and thujene were affected by temperature as well as light intensity in beech (*Fagus sylvatica* L.) [9]. Similarly, egg deposition by the pine sawfly (*Diprion pini*) induced the emission of mono- and sesquiterpenes from Scots pine (*Pinus sylvestris*) needles [11]. In addition, the production and emission of terpenoids are also regulated in a tissue- and/or organ-specific manner. Genes involved in the biosynthesis of terpenoids are expressed at particular stages of plant development and/or in specific tissues [13,14,15,16,17]. 

Stevia (*Stevia rebaudiana*), a perennial shrub belonging to Asteraceae (2n = 22), is appreciated worldwide for its sweetness. Sweetness in Stevia comes from diterpenoid steviol glycosides (SGs), which are about 300 times sweeter than sugar. Thus, they are widely used in the food industry as a sugar substitute and sweetener [18]. In addition to SGs, Stevia tissues also produce diverse terpenoids [16,19]. Aerial parts of Stevia plants grown in Italy were shown to produce mainly mono- and sesquiterpenoids [19]. Trichomes isolated from Stevia leaves accumulated high amounts of mono-, sesqui-, and diterpenoids, along with minor quantities of fatty acid derivatives [16].

So far, studies in Stevia have focused mainly on types and accumulation of diterpenoid SGs, their biosynthetic genes, and pathways. In contrast, research efforts related to profiling and biosynthesis of other chemicals are largely missing in Stevia. Here, we showed comprehensive profiles of terpenoids in four types of Stevia tissues—leaves, flowers, stems, and roots. From the transcriptome data of Stevia, we identified four sesqui-TPSs and one mono-TPS, which were later found to be responsible for the production of major terpenoids in Stevia tissues via biochemical assays. Furthermore, our study identified two root-specific, multifunctional sesqui-TPSs that were capable of catalyzing multiple products, γ-curcumene/zingiberene/β-sesquiphellandrene and α-longipinene/β-himachalene/himachalol. We also found that most *SrTPSs* are highly responsive to environmental stresses showing different inducibility in leaves and roots of Stevia. These results suggest that Stevia could use terpenoids produced by these SrTPSs in each tissue for the defense mechanism against biotic and abiotic stresses.

## 2. Results

### 2.1. Analysis of Terpenoids in Stevia

**Figure 1:**
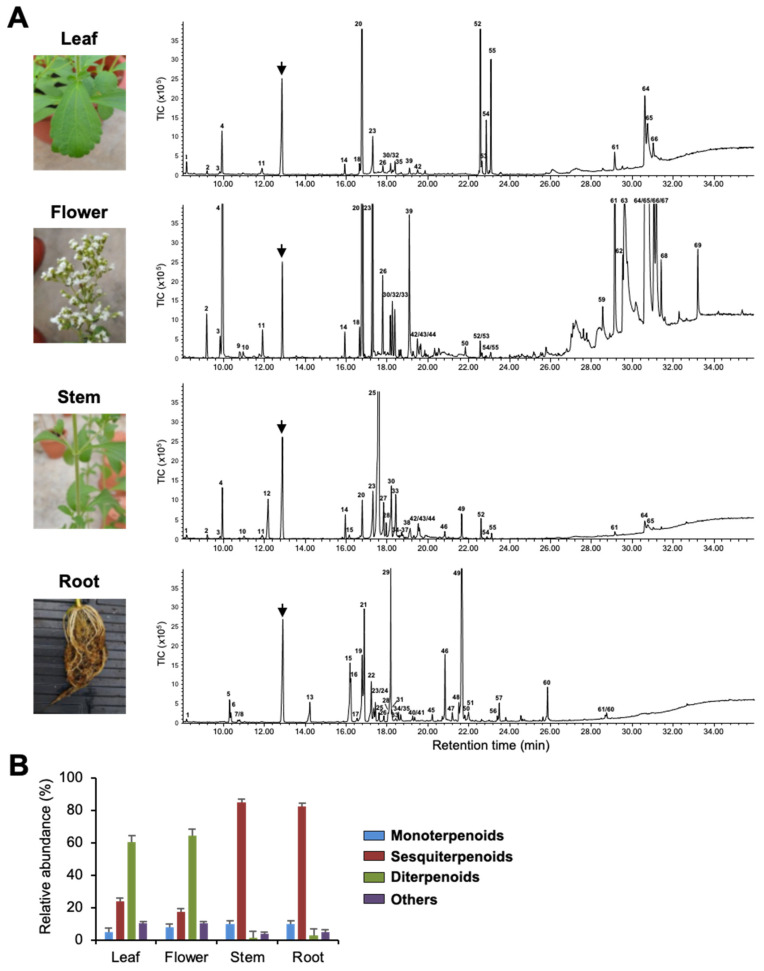
Volatile organic compounds (VOCs) of Stevia tissues. (**A**) Photographs of Stevia leaves, flowers, stems, and roots and their VOCs’ emission profile shown by gas chromatogram. The arrows indicate the internal standard camphor. Peaks numbers are identical to those listed in Table 1. TIC, Total ion chromatogram. (**B**) Classification of Stevia VOCs. Data are the mean ± standard deviation of three readings.

To investigate terpenoids produced by Stevia tissues, we extracted volatiles from leaves, flowers, stems, and roots and analyzed them by Gas Chromatography–Mass Spectrometry (GC-MS). Figure 1, Supplementary Figure S1, and Table 1 show qualitative and quantitative variation in the composition of VOCs in the four tissues. Flowers and roots produced 36 compounds each, whereas stems and leaves produced a total of 34 and 23 compounds, respectively. Among all tissues, flowers had the highest number of VOCs, followed by leaves and roots, with stems producing the least quantity (Supplementary Figure S1). Among the putatively identified VOCs, terpenoids were the predominant class of volatiles in all four tissues and contributed more than 90% to total VOCs’ composition. Among terpenoids, diterpenoids were mainly found in leaves and flowers, whereas sesquiterpenoids dominated the volatile compositions of stems and roots (Figure 1B). Stevia flowers produced large quantities of labdane-type diterpenoids, such as copalic acid, copaiferic acid, and 8(17), 12-labda-diene-15,16-dial (Figure 1 and Table 1). Even though copalic acid and copaiferic acid were detected in leaves, the levels were lower than those of flowers. Unlike flowers and leaves, stems, and roots accumulated several sesquiterpenoids accounting for more than 80% of total VOCs (Figure 1B, Table 1). β-farnesene was the most abundant compound produced by stems (52.53%), whereas roots possessed large quantities of himachalol (21.00%), β-sesquiphellandrene (15.91%), α/β-isocomene (14.90%), and α-longipinene (3.18%) (Figure 1A and Table 1). 

Of the 60 terpenoids identified, only three sesquiterpenoids (β-caryophyllene, α-caryophyllene, and bicyclogermacrene) were found in all four tissues, where they showed variation in quantities (Table 1). In addition, we observed tissue-specificity in the production of a few terpenoids. For instance, α-terpeniol, α-bergamotol, γ-bisabolene, and β-caryophyllene oxide could only be detected in stems, while limonene was found only in flowers. Interestingly, out of 30 terpenoids produced by Stevia roots, 18 were found only in roots. Tissue-specific emission of these terpenoids might be essential for the interaction of roots with beneficial soil microbes and/or for the protection of roots from pathogens (Table 1).

### 2.2. Identification of Terpene Synthases from Stevia

Terpenoids were the major VOCs in all four tissues studied (Figure 1 and Table 1). From Stevia RNA-seq data [13], we were able to find five full-length open reading frames (ORFs) of mono- or sesqui-TPSs, which were later designated as *SrTPS1*-*5*. Phylogenetic analysis showed that five SrTPSs were grouped into TPS-a and TPS-b subfamilies (Supplementary Figure S2). TPS-a and TPS-b subfamilies are known to include TPSs for the biosynthesis of sesquiterpenoids and monoterpenoids, respectively [7]. Deduced amino acid sequences of *SrTPS1-5* contained typical motifs of TPS enzymes. DDXXD and NSE/DTE that are required for binding of substrates and cofactors were conserved in all SrTPSs (Supplementary Figure S3). Another conserved motif, R(R)X8W, which plays a role in the complexation of the pyrophosphate group after ionization of the substrate, was found in all SrTPSs. ChloroP analysis showed that among SrTPSs, SrTPS1 contained 30 amino acid plastidial transit peptide (Tp) sequence at N-terminus.

**Figure 2:**
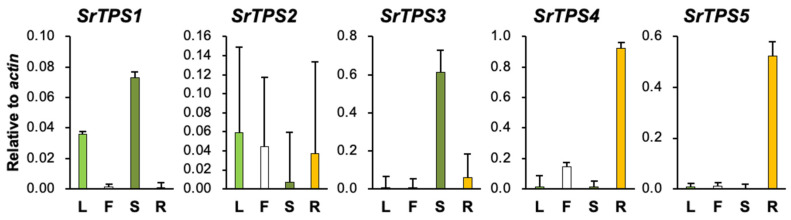
qRT-PCR analysis of *SrTPS* genes in different tissues. L, leaf; F, flower; S, stem, and R, root. The housekeeping gene *actin* was used as control. Data are the mean ± standard deviation of three readings.

The relative expression levels of five *SrTPSs* in four Stevia tissues were examined by qRT-PCR (Figure 2). *SrTPS2* did not show much variation in expression levels among the four tissues. *SrTPS1* and *SrTPS3* were abundant in stems, whereas *SrTPS4* and *SrTPS5* showed the highest expression in roots with little or no expression in other tissues (Figure 2). 

**Figure 3:**
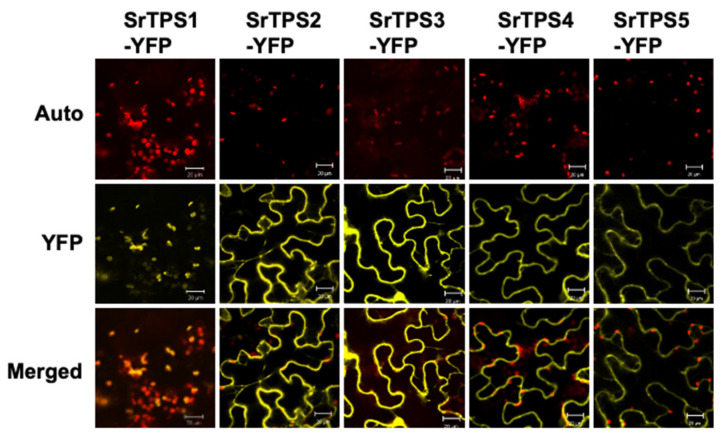
Subcellular localization of Stevia terpene synthases (TPSs). Subcellular localization of SrTPSs fused with YFP in *N. benthamiana* leaves. The *Agrobacterium*-infiltrated leaves were visualized using confocal microscopy. Auto, chlorophyll auto-fluorescence; YFP, YFP channel image; Merged, merged image of Auto and YFP. Scale bars, 20 μm.

To observe the subcellular localization of SrTPSs, we transiently expressed the full-length ORF of each *SrTPS* fused with the *YFP* gene in *Nicotiana benthamiana* leaves using *Agrobacterium*-mediated infiltration. Figure 3 shows that YFP-fused SrTPS2-5 were localized in the cytosol, where they might be associated with the production of sesquiterpenoids. On the other hand, SrTPS1 was localized in chloroplasts, indicating that it might be a mono-TPS.

### 2.3. Functional Characterization of SrTPSs

**Figure 4:**
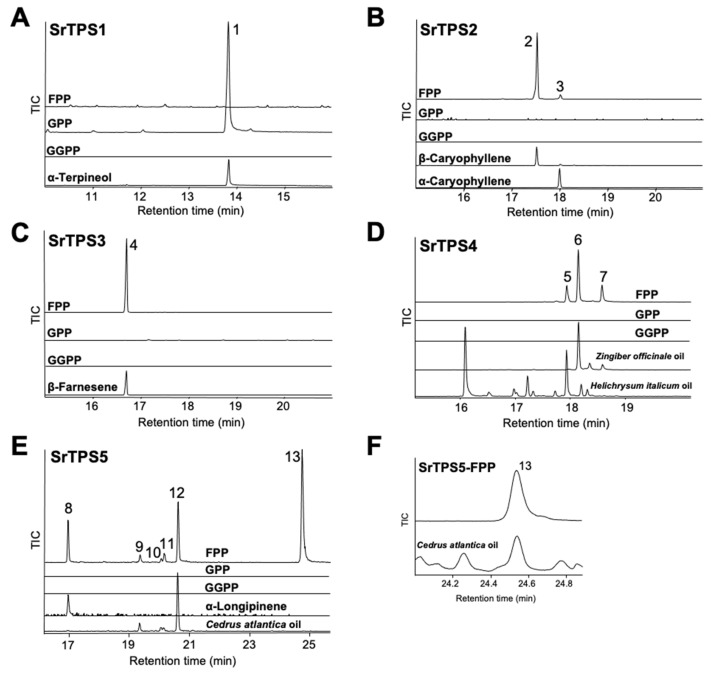
In vitro characterization of five Stevia TPSs. Gas chromatograms of products obtained from in vitro enzymatic assay of (**A**) SrTPS1, (**B**) SrTPS2, (**C**) SrTPS3, (**D**) SrTPS4, and (**E**) SrTPS5. (**F**) Alignment of peak 13 with himachalol from the essential oil of *Cedrus atlantica*. Purified recombinant proteins were incubated with the substrates geranyl pyrophosphate (GPP), farnesyl pyrophosphate (FPP), and geranylgeranyl pyrophosphate (GGPP). Peaks 1, 2, 3, 4, and 8 were identified using authentic standards. The essential oil of *Helichrysum italicum* was used as standard for the identification of peak 5, whereas peaks 6 and 7 were verified using the *Zingiber officinale* (ginger) essential oil. For validation of peaks 9, 10, 12, and 13, the essential oil from *Cedrus atlantica* was used. For peak 11, National Institute of Standards and Technology MS 2014 library was used. 1, α-terpineol; 2, β-caryophyllene; 3, α-caryophyllene; 4, β-farnesene; 5, γ-curcumene; 6, zingiberene; 7, β-sesquiphellandrene; 8, α-longipinene; 9, α-himachalene; 10, γ-himachalene; 11, 10s,11s-himachala-3(12),4-diene; 12, β-himachalene; 13, himachalol. Mass spectra of all the compounds are given in Supplementary Figure S4. TIC, Total ion chromatogram.

To elucidate the exact function of SrTPSs, full-length ORFs of *SrTPS*s were expressed as recombinant proteins in *Escherichia coli* C41 (DE3) cells. Purified recombinant proteins were then tested for activity against geranyl pyrophosphate (GPP), farnesyl pyrophosphate (FPP), and geranylgeranyl pyrophosphate (GGPP), and products were analyzed by GC-MS. Figure 4 shows that SrTPS2-5 reacted only with FPP to produce sesquiterpenoids, whereas SrTPS1 reacted with GPP alone to produce a monoterpenoid. These results were consistent with our prediction on the potential function of SrTPSs based on phylogenetic analysis and subcellular localization experiments. SrTPS1, as a member of the TPS-b family, utilized only GPP to synthesize α-terpineol (Figure 4A). The major product of SrTPS2 was β-caryophyllene, along with minor amounts of α-caryophyllene (Figure 4B). SrTPS3 produced β-farnesene as a single product, which was the most abundant volatile compound in stems (Figure 4C and Table 1). SrTPS4 catalyzed the formation of three compounds, γ-curcumene, zingiberene, and β-sesquiphellandrene (Figure 4D). SrTPS5 predominantly formed himachalol, β-himachalene, and α-longipinene, along with minor amounts of α-himachalene, γ-himachalene, and 10s,11s-himachala-3(12),4-diene from FPP (Figure 4E,F). No product was observed from the heat-inactivated SrTPSs, which served as negative controls (Supplementary Figure S5). The products produced by SrTPSs were verified using authentic standards and essential oils that contain the terpenes of our interest. 

**Figure 5:**
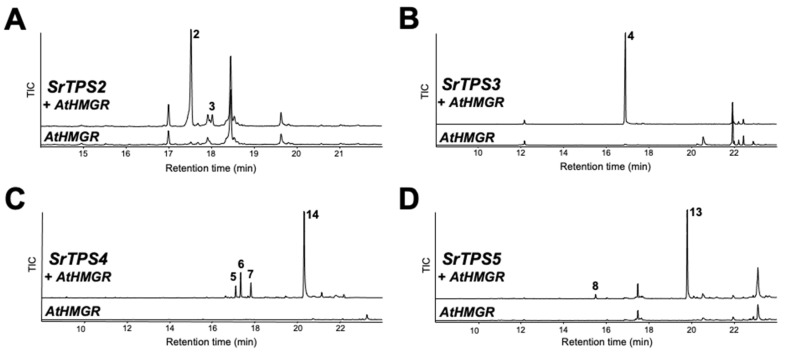
In vivo characterization of four Stevia TPSs. Gas chromatograms of extracts obtained from leaves of *N. benthamiana* plants transiently overexpressing *AtHMGR* along with (**A**) *SrTPS2*, (**B**) *SrTPS3*, (**C**) *SrTPS4,* and (**D**) *SrTPS5*. Plants overexpressing *AtHMGR* alone was used as control. 2, β-caryophyllene; 3, α-caryophyllene; 4, β-farnesene; 5, γ-curcumene; 6, zingiberene; 7, β-sesquiphellandrene; 8, α-longipinene; 13, himachalol; 14, zingiberenol. Mass spectra of the compounds are given in Supplementary Figure S4. TIC, Total ion chromatogram.

The terpenoid profiles of SrTPSs obtained by in vitro analysis were verified by transiently expressing them in *N. benthamiana* leaves using *Agrobacterium*-mediated infiltration. The Arabidopsis *3-hydroxy-3-methylglutaryl coenzyme A reductase* (*HMGR*) was co-expressed with sesqui-TPSs to increase heterologous sesquiterpenoid production in *N. benthamiana,* and its effects have been confirmed before [20]. GC-MS analysis of leaves expressing *SrTPS2* and *SrTPS3* revealed the presence of α- and β-caryophyllene and β-farnesene, respectively, mimicking the in vitro results (Figure 5A,B). However, SrTPS4 and SrTPS5 yielded slightly different products in vivo compared to their corresponding in vitro assays. Plants expressing *SrTPS4* produced zingiberenol (peak 14), in addition to three compounds identified from in vitro assay (Figure 5C). On the other hand, only α-longipinene and himachalol could be detected from *N. benthamiana* expressing *SrTPS5* (Figure 5D). Although we identified SrTPS1 as α-terpineol synthase by in vitro assay, α-terpineol could not be detected from *N. benthamiana* plants transiently overexpressing *SrTPS1*, suggesting the extreme instability of α-terpineol in mesophyll cells of *N. benthamiana* or possible further metabolism in *N. benthamiana* plants.

### 2.4. Expression of SrTPSs under Multiple Stress Conditions

**Figure 6:**
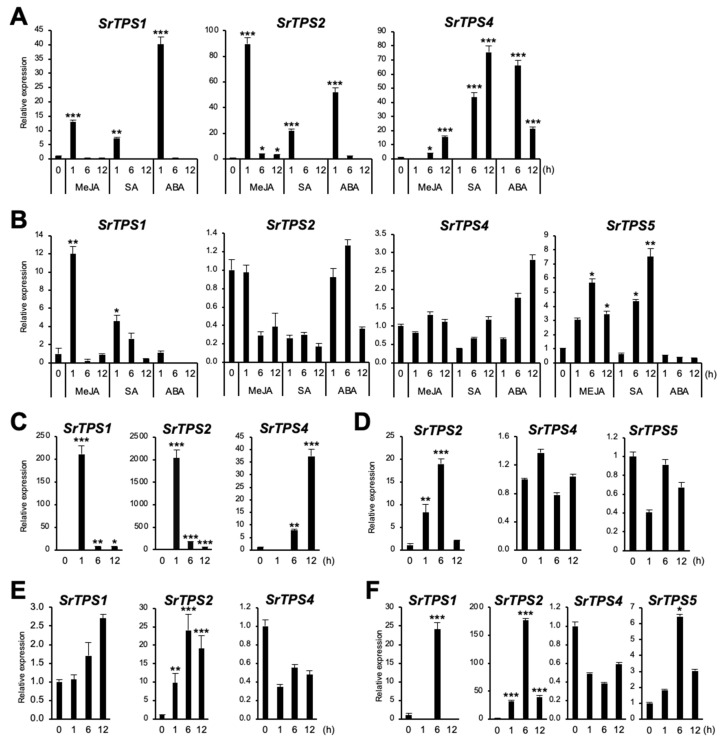
Expression levels of Stevia *TPS*s under various environmental stresses. (**A**,**B**) hormone treatments, (**C**,**D**) wounding, (**E**,**F**) dehydration, (**A**,**C**,**E**) leaf, (**B**,**D**,**F**) root. MeJA, methyl jasmonate; SA, salicylic acid; ABA, abscisic acid. The housekeeping gene *actin* was used for normalization. Data are the mean ± standard deviation of three readings. Statistical significance of the measurements was determined by Student’s *t*-test (* *p* < 0.05, ** *p* < 0.01, *** *p* < 0.005).

In general, plant *TPSs* are induced by environmental stresses [21]. To investigate if *SrTPSs* are responsive to different environmental stresses, stress-related phytohormones, such as methyl jasmonate (MeJA), salicylic acid (SA), and abscisic acid (ABA), as well as wounding and dehydration stresses were applied. Figure 6A shows that *SrTPS1* and *SrTPS2* exhibited an early response to MeJA, SA, and ABA in leaves, whereas *SrTPS4* was gradually induced under MeJA and SA treatment and reached its highest at 6 h of ABA treatment. In the roots of Stevia, when *SrTPS2* remained nearly unaltered or slightly decreased upon all phytohormone treatment, *SrTPS4* was gradually induced only by ABA but not by MeJA and SA (Figure 6B). On the other hand, *SrTPS1* and *SrTPS5* transcripts were induced early and slightly late by MeJA and SA, respectively (Figure 6B). Note that *SrTPS3* and *SrTPS5* in leaves were omitted due to undetectable levels of transcripts by phytohormone treatments, and *SrTPS3* in roots was not responsive to any phytohormone treatment. 

Similar results were obtained for *SrTPS1*, *SrTPS2,* and *SrTPS4* in wounded leaves (Figure 6C). However, in roots, we were only able to find *SrTPS2* induced by wounding (Figure 6D). The level of *SrTPS4* and *SrTPS5* transcripts remained unchanged, and others were very low levels or not detectable in roots. 

Under dehydration, the levels of *SrTPS1* and *SrTPS2* in leaves gradually increased and stayed high at 12 h (Figure 6E). Interestingly, the transcript levels of *SrTPS1*, *SrTPS2,* and *SrTPS5* were highest at 6 h in roots. Significantly, the induction levels of *SrTPS1* and *SrTPS2* were more than 20 and 160 times under dehydration conditions, respectively (Figure 6F). Unlike other *SrTPSs*, the expression of *SrTPS4* was slightly downregulated by dehydration in both leaves and roots (Figure 6E,F). 

All conditions for stress treatments in this study were verified by the expression of homologs of Arabidopsis stress-induced marker genes, such as *Ethylene-Responsive element-binding Factor 1* (*ERF1*) for wounding and MeJA [22], *Glutathione S-Transferase 6* (*GST6*) for SA [23], and *Responsive to ABA 18* (*RAB18*) for dehydration and ABA [24,25], which were highly upregulated under the above-mentioned stress conditions (Supplementary Figure S6). 

## 3. Discussion

Most of the studies using Stevia have focused on diterpenoid SGs due to its commercial importance, while no research has investigated the biosynthesis of other terpenoids in Stevia. From our VOC analysis, we found the following results regarding terpenoids biosynthesis in Stevia. (1) Over 90% of the total VOCs of Stevia leaves, flowers, stems, and roots were composed of terpenoids. (2) Diterpenoids were mainly accumulated in Stevia flowers and leaves. (3) Sesquiterpenoids were the major VOCs of roots and stems. (4) Many terpenoids were differentially produced in Stevia tissues. (5) Most terpenoids identified from roots were root-specific (Figure 1 and Table 1). However, information to uncover biosynthetic pathway genes for terpenoids other than SGs was limited in Stevia until this study. 

Through the search for full-length mono- and sesqui-TPSs and biochemical assays, we were able to characterize five SrTPSs. SrTPS1 was a mono-TPS catalyzing the formation of α-terpineol from GPP. Terpineol, a mixture of four isomers, α-, β-, γ-terpineol, and terpinen-4-ol, is known for its pleasant fragrance. Some terpineol synthases can produce multiple enantiomeric forms [26,27] while others produce a single product similar to SrTPS1 [28,29]. *SrTPS1* was highly responsive to most phytohormones and stresses in leaves and roots (Figure 6). Since α-terpineol is shown to be toxic to insects and pests [30], SrTPS1 may play a crucial role in plants’ defenses. 

BLASTP analysis showed that SrTPS2 had the highest similarity to the β-caryophyllene synthase (78% identity) from *Artemisia annua* [31]. Interestingly, SrTPS2 could produce β-caryophyllene and its isomer, α-caryophyllene in vitro, as well as in planta. The expression pattern of *SrTPS2* was comparable in all four tissues, which was in correlation with the levels of α- and β-caryophyllenes (Figure 1A and Figure 2, and Table 1). *SrTPS2* transcript was quickly induced upon MeJA and wounding treatments in leaves (Figure 6A,C), confirming its defense roles against herbivore or pathogens [32,33]. Moreover, *SrTPS2* was highly responsive to dehydration and wounding stresses in roots (Figure 6D,F). These results suggest that α/β-caryophyllenes may be released in response to biotic and abiotic stresses in both above and below ground Stevia tissues. Intriguingly, α/β-caryophyllenes were relatively abundant in flowers compared to other tissues (Table 1). β-caryophyllene was reported to be a main constituent of the volatiles in aerial parts to attract pollinators [34,35]. Thus, Stevia may use β- and/or α-caryophyllene as one constituent of volatiles for pollinator attraction.

β-farnesene was one of the most abundant VOCs in Stevia stems (Figure 1A and Table 1). We demonstrated SrTPS3 to be the key enzyme behind its production (Figure 4C and Figure 5B). The levels of β-farnesene were consistent with the predominant expression of *SrTPS3* in stems (Figure 2). Interestingly, transcript levels of *SrTPS3* remained unaffected in Stevia leaves and roots even after exposure to phytohormones, wounding, and dehydration stresses, indicating that it is a stem-specific TPS in Stevia. SrTPS3 may protect Stevia stems from aphids as β-farnesene is the major component of the aphid alarm pheromone [36].

SrTPS4 showing the highest homology (74% identity) with (Z)-γ-bisabolene synthase from *Helianthus annuus* catalyzed the formation of multiple sesquiterpenoids, γ-curcumene, zingiberene, and β-sesquiphellandrene, upon reaction with FPP (Figure 4D). These reaction products could also be found among the compounds produced in vitro by zingiberene synthases in many plants, including tomato and rice, suggesting functional similarity [37,38]. As zingiberene and β-sesquiphellandrene were the most abundant terpenes in ginger and turmeric, they are among the most important sesquiterpenoids for the defense in roots [37,39]. Although zingiberenol was not produced by SrTPS4 in vitro, the transient expression of *SrTPS4* in *N. benthamiana* formed zingiberenol in addition to γ-curcumene, zingiberene, and β-sesquiphellandrene (Figure 5C). Moreover, we also detected zingiberenol among Stevia root VOCs (Table 1), suggesting a possible further modification of zingerberene in Stevia and *N. benthamiana* by enzymes, such as cytochrome P450s. Zingiberene, γ-curcumene, and β-sesquiphellandrene can be formed together from (6R,7S)-bisabolyl cation via enzymatic cyclization of FPP [40]. Interestingly, environmental stresses tested in this study did not increase transcript levels of *SrTPS4* in Stevia roots, where it is highly expressed under normal conditions. However, its expression was induced in leaves upon phytohormone treatments and wounding stress (Figure 6A,C). 

Purified recombinant SrTPS5 produced mainly himachalol and β-himachalene with minor amounts of α-longipinene, α-himachalene, γ-himachalene, and 10s,11s-himachala-3(12),4-diene from FPP (Figure 4E,F). Until now, TPSs producing α-longipinene have only been reported in gymnosperms as multifunctional TPSs [41,42,43]. PaTPS-Lon from Norway spruce produced α-longipinene with other sesquiterpenoids, longifolene, α-longicyclene, β-farnesene, and longiborneol [41]. Similarly, in addition to α-longipinene, PsTPS-Lonp from Sitka spruce (*Picea sitchensis*) was also able to form substantial amounts of longifolene, γ-himachalene, and β-farnesene [43]. Himachalol was identified as a primary component in the essential oil of many plants, including *Chromolaena odorata* [44], *Cedrus atlantica* [45], and *Inula britannica* [46]. *MtTPS10* from *Medicago trancatula* was reported to be involved in the biosynthesis of himachalol [47]. 

Products of SrTPS5 contributed nearly 25% to the total Stevia root VOCs implying that it may have important ecological roles in aiding roots’ responses to belowground biotic and abiotic factors. α-longipinene was predicted to play a role in defense as its level increased in Sitka spruce stems upon weevil attack [48], while himachalol and himachalenes possessed insecticidal and larvicidal properties [49,50]. It should be noted that the induction of *SrTPS5* by MeJA, SA, and dehydration treatments was root-specific. Overall, Stevia is likely to express *SrTPS5* for belowground defense strategies. 

In conclusion, we have shown SrTPSs that are responsible for the biosynthesis of major sesqui- and monoterpenoids in Stevia tissues. The terpene profiles of Stevia tissues could be explained by activities of both single-product and multi-product SrTPSs. The ability of SrTPS4 and SrTPS5 to synthesize more than one product from a single substrate may provide Stevia with access to variable chemical defense in response to herbivores and/or pathogens. Future studies will be directed towards a better understanding of the ecological roles of these terpenoids in aerial as well as belowground tissues of Stevia. 

## 4. Materials and Methods 

### 4.1. Plant Materials

Stevia and *N. benthamiana* seeds were sown in a potting soil mixed with sand and grown in a greenhouse under natural light conditions (12 h light/12 h dark) in Singapore (1.29°N, 103.77°E). Singapore has a typical tropical climate, high and uniform temperatures, and high humidity all year round (http://www.weather.gov.sg/climate-climate-of-singapore/). Once the seeds germinated, the seedlings were then transferred to bigger pots with potting soil and sand and covered for 3 days with a transparent plastic dome for hardening. All greenhouse plants were watered every three days. Four-week-old *N. benthamiana* plants were used for subcellular localization studies and in vivo characterization of SrTPSs.

Leaves, flowers, stems, and roots were collected at the same time from three-month-old Stevia plants and frozen in liquid nitrogen. The frozen samples were immediately processed for volatile analysis and gene expression studies. 

For stress assays, Stevia seeds were germinated on Murashige and Skoog (MS) medium with 6.5 g/L agar and 0.5 mg/L of indole-3-acetic-acid and propagated on fresh media every 3–4 weeks. The in vitro plants were kept in a plant growth chamber and maintained at 25 °C, 16 h L/8 h D. Hormone treatments were carried out by soaking 3-week-old in vitro plants in 30 mL MS media containing one of the following: 50 μM MeJA, 50 μM ABA, or 100 μM SA. For wounding the leaves, 1.5 mm diameter holes were punched in the youngest, fully opened leaf using a multiple hole puncher. Roots were wounded by cutting them every 3–4 mm, and the cut roots were placed in MS media. For dehydration assay, whole plants were left to dry on a laboratory bench at 25 °C. Leaf and root samples were harvested from stress-treated plants at 1, 6, and 12 h and frozen in liquid nitrogen. The frozen samples were immediately processed for RNA isolation. 

### 4.2. Extraction of Essential Oils from Stevia Tissues

For VOCs extraction, frozen Stevia leaves, flowers, stems, and roots were ground using a pre-chilled mortar and pestle, and 500 mg of frozen crushed plant crystals was resuspended in 500 μL of ethyl acetate (ThermoFisher Scientific, Waltham, MA, USA). Camphor (10 µg/µL) was added as an internal standard. The mixture was incubated on a horizontal shaker at 200 rpm for 2 h at 25 °C. After centrifugation of the mixture at 4000 rpm for 20 min at 4 °C, the upper layer was transferred into a 2 mL vial and dehydrated using anhydrous sodium sulfate (Sigma–Aldrich, St. Louis, MO, USA). After a brief centrifugation, the extract was transferred into a fresh vial, and 1 μL was injected into GC-MS. 

### 4.3. RNA Isolation, cDNA Synthesis and Quantitative Real-Time PCR (qRT-PCR)

Total RNA was extracted from frozen crushed crystals of Stevia leaves, flowers, stems, and roots using the Spectrum™ Plant Total RNA Kit (Sigma–Aldrich, St. Louis, MO, USA) according to manufacturer instructions. On column RNase-free DNase I (Qiagen, Hilden, Germany), treatment was carried out to remove the residual genomic DNA. Total RNA was quantified using a NanoDrop 2000 spectrophotometer (Thermo Fisher Scientific, Waltham, MA, USA) and stored at −80 °C until use. 

Complementary DNA (cDNA) was synthesized from 1 µg of the total RNA using M-MLV reverse transcriptase (Promega, Madison, WI, USA), dNTP, and oligo (dT) primer according to manufacturer instructions. cDNA was diluted with RNase free water to a final concentration of 10 ng/µL and stored at −20 °C until use.

qRT-PCR was performed using an Applied Biosystems 7900HT fast real-time PCR system and TAKARA SYBR Premix Ex Taq II (TaKaRa, Kusatsu, Japan). Primers for qRT-PCR were designed using a Primer3 program (http://bioinfo.ut.ee/primer3-0.4.0/) and are listed in Supplementary Table S1. PCR primer efficiency was determined using four serial cDNA dilutions (1:1, 1:5, 1:25, and 1:125) and the equation (%) = (10^(−1/slope)^−1) × 100) [51]. Primer pairs with efficiency values between 90 and 110% were chosen. Stevia *actin* gene was used as an internal control for data normalization. Relative quantitation by RT-PCR was performed in a 10 µL volume containing 1 µL of cDNA, 5 µL of 2 x TAKARA SYBR Premix Ex Taq II, 0.5 µL of 10 µM forward primer, 0.5 µL of 10 µM reverse primer, and 3 µL of water. The plate was covered using a microseal ‘B’ seal (Bio-Rad, Hercules, CA, USA) for optical transparency and centrifuged briefly using a PCR plate spinner (VWR, Radnor, PA, USA). The plate was subjected to the following cycling program: 3 min at 95 °C, followed by 40 cycles of 10 s at 95 °C and 30 s at 60 °C; then final ramping to 95 °C at the rate of 0.5 °C/5 s for melting curve analysis. For verification of a single product amplification, both melting curve analysis and gel electrophoresis were used. Non-template control and non-RTase treated templates were included to detect and eradicate primer–dimer formation and genomic DNA contamination. All qRT-PCR experiments were carried out in three technical replicates of two biological replicates. SDS 2.4 (Applied Biosystems, Waltham, MA, USA) was used to analyze the results. For the relative expression of *SrTPSs* among different tissues, comparative dCt values of target genes to *actin* were calculated by 2^−(dCt)^ where dCt = Ct,*target* − Ct,*actin*. For stress assays, data were analyzed using 2^−(ddCt)^ where ddCt = (Ct,*target* − Ct,*actin*)Time x − (Ct,*target* − Ct,*actin*)Time 0. Time x represents the treatment duration and Time 0 represents the untreated control [52]. 

### 4.4. Isolation of Full-Length ORF of Stevia Genes and Vector Construction

The full-length ORFs of SrTPS1-5 were amplified from cDNA of different Stevia tissues using iProof™ High-Fidelity DNA Polymerase (Bio-Rad, Hercules, CA, USA). The primers are listed in Supplementary Table S1. The amplified gene products were cloned into a Gateway pDONR221 vector using BP clonase and transformed into One Shot TOP10 competent cells (Invitrogen, Carlsbad, CA, USA). The positive clones were validated by sequencing. For purification of GST- or 6His-tagged recombinant protein from *E. coli*, the pDONR221 clone harboring each gene was inserted into either pDEST15 or pDEST17 destination vectors, respectively by LR Clonase (Invitrogen, Carlsbad, CA, USA). For plant expression, the pDONR221 clones harboring genes of interest were inserted into the destination vector, pBA-DC-YFP expression vector, which contained a cauliflower mosaic virus 35S promoter (*CaMV 35S*) and a C terminus in frame with yellow fluorescent protein (*YFP*) gene by LR Clonase (Invitrogen, Carlsbad, CA, USA). 

### 4.5. Sequence Alignment and Phylogenetic Analysis

The presence of chloroplast signal peptide was predicted using ChloroP (http://www.cbs.dtu.dk/services/ChloroP). Multiple sequence alignment of deduced amino acid sequences was constructed with CLUSTALW using default parameters. Multiple sequence alignment was carried out using CLUSTALW, and the evolutionary history was inferred by using the Maximum Likelihood method [53]. The tree with the highest log likelihood (−48972.54) is shown. The percentage of trees in which the associated taxa clustered together is shown next to the branches. Initial tree(s) for the heuristic search were obtained automatically by applying Neighbor-Joining (NJ) and BioNJ algorithms to a matrix of pairwise distances estimated using the Jones–Taylor–Thornton model, and then selecting the topology with superior log likelihood value. A discrete gamma distribution was used to model evolutionary rate differences among sites (5 categories (+*G*, parameter = 2.6411)). The tree is drawn to scale, with branch lengths measured in the number of substitutions per site. The analysis involved 46 amino acid sequences. All positions containing gaps and missing data were eliminated. There was a total of 1042 positions in the final dataset. Evolutionary analyses were conducted in MEGA X [54]. Abbreviations and GenBank accession numbers of proteins used in phylogenetic trees are listed in Supplementary Table S2.

### 4.6. Subcellular Localization and In Vivo Assay

Plasmids harboring *SrTPS1-YFP*, *SrTPS2-YFP*, *SrTPS3-YFP*, *SrTPS4*-*YFP,* and *SrTPS5-YFP* constructs were transformed into *Agrobacterium tumefaciens* GV3101 strain by electroporation and grown on Luria Bertani (LB) plates containing 20 mg/L of rifampicin and 25 mg/L of spectinomycin. 

Cultures obtained from the above transformation were infiltrated into 4-week-old *N. benthamiana* leaves using a needleless 1 mL syringe. The agro-infiltrated plants were maintained in long-day conditions (16 h light/8 h dark, 25 °C).

For subcellular localization, infiltrated *N. benthamiana* leaves were mounted on slides three days post infiltration (dpi) and imaged using an LSM5 Exciter (Carl Zeiss, Oberkochen, Germany) confocal scanning laser microscope with a standard filter set. Images were processed using an LSM Image Browser (Carl Zeiss, Oberkochen, Germany). 

For in vivo characterization of SrTPSs, VOCs were collected from *N. benthamiana* leaves 3 dpi, as described in the extraction of essential oils from Stevia tissues.

### 4.7. In Vitro TPS Assay

For heterologous expression of *SrTPSs*, pDEST15, and pDEST17 vectors containing *SrTPS* genes were transformed into *E. coli* C41(DE3) and grown on LB agar plates containing 100 mg/L of ampicillin. Zero-point four millimolar isopropyl-β-D-thiogalactoside (IPTG) was added to induce the expression of fusion proteins in bacterial cells at 25 °C for 6 h. His-tagged SrTPS2 and SrTPS3 were purified using Ni-NTA Sepharose resin (Qiagen, Hilden, Germany), whereas GST-tagged SrTPS1, SrTPS4, and SrTPS5 were purified by using glutathione–agarose resin (Sigma–Aldrich, St. Louis, MO, USA) according to the manufacturer’s recommendations. The purified proteins were immediately used for in vitro TPS assay. An in vitro enzyme assay for TPS activity was performed in a 500 µL reaction volume containing 250 μL of 2 × reaction buffer specific for monoterpene (50 mM HEPES, pH 7.4, 200 mM KCl, 20 mM MnCl_2_, 20% (*v/v*) glycerol, 5 mM dithiothreitol) or sesquiterpene (50 mM HEPES, pH 7.4, 200 mM KCl, 20 mM MgCl_2_, 20% (*v/v*) glycerol, 2 mM dithiothreitol) biosynthesis with about 20 μg of recombinant protein and 10 μg of GPP or FPP (Echelon Biosciences, Salt Lake City, UT, USA). The reaction mixtures were mixed gently and carefully overlaid with 500 μL of hexane (Sigma–Aldrich, St. Louis, MO, USA) to trap volatile products and incubated at 30 °C for 2 h. As negative controls, the heat-inactivated recombinant proteins were tested. After centrifugation at 1200× *g* at 4 °C for 30 min, the hexane upper layer was concentrated to 50 μL using nitrogen gas evaporator and analyzed by GC-MS. 

### 4.8. GC-MS Analysis

VOCs were analyzed by an Agilent 7890A GC, coupled with a 5975C inert mass selective detector (Agilent Technologies, Santa Clara, CA, USA). An autosampler was used to inject the samples in splitless mode into a port heated to 250 °C and an oven heated to 50 °C. Separation was achieved using an HP-5MS column (30 m × 0.25 mm × 0.25 µm) with helium carrier gas at a constant flow rate of 1 mL/min. The GC oven temperature was programmed from 50 °C (held for 1 min) to 300 °C at 8 °C/min and finally held at 300 °C for 5 min. MS measurements were performed in the scan mode with the scan range of m/z 50 to 350. C_7–_C_30_ saturated alkanes were used for the calculation of retention indices. The MSD ChemStation data analysis program (Agilent Technologies, Santa Clara, CA, USA) was used for data processing. For VOCs analysis, peaks were identified by comparison of retention times, retention indices, and mass spectra with those from the National Institute of Standards and Technology (NIST) MS 2014 library. Peak areas of individual products were calculated as follows. Product (%) = (peak area of the product/sum of peak areas of all the products produced by a tissue) × 100. 

For in vitro and in vivo analysis, peaks were identified by the comparison of retention times, retention indices, and mass spectra with entries in the NIST MS 2014 library and/or authentic standards. Authentic standards of α-terpineol, β-caryophyllene, α-caryophyllene, β-farnesene, and α-longipinene were purchased (Sigma–Aldrich, St. Louis, MO, USA). Essential oils of *Cedrus atlantica*, *Helichrysum italicum,* and *Zingiber officinale* were procured (Organic Infusions Inc., Camarillo, CA, USA). The suppliers of the essential oils provided the list of all volatile compounds with their retention times and quantities. 

## Figures and Tables

**Table 1:** Volatile organic compounds (VOCs) of Stevia tissues.

No. ^a^	Compound name	RT ^b^	RI ^c^	Molecular Formula	RA (%) ^d^			
					L	F	S	R
1	p-Xylene	8.17	886	C_8_H_10_	1.10		0.29	0.20
2	α-Pinene	9.18	962	C_10_H_16_	0.27	0.51	0.28	
3	β-Phellandrene	9.81	1009	C_10_H_16_	0.28	0.35	0.19	
4	β-Pinene	9.97	1021	C_10_H_16_	3.52	5.73	3.59	
5	α-Terpinolene	10.27	1044	C_10_H_16_				1.81
6	α-Phellandrene	10.34	1049	C_10_H_16_				0.82
7	β-Cymene	10.68	1074	C_10_H_14_				0.26
8	D-Sylvestrene	10.76	1080	C_10_H_16_				0.27
9	Limonene	10.80	1084	C_10_H_16_		0.30		
10	β-Ocimene	10.98	1096	C_10_H_16_		0.20	0.37	
11	β-Linalool	11.93	1168	C_10_H_18_O	0.89	0.37	0.54	
12	α-Terpineol	12.17	1186	C_10_H_18_O			4.51	
13	O-Methylthymol	14.19	1338	C_11_H_16_O				2.59
14	γ-Elemene	15.97	1472	C_15_H_24_	0.87	0.26	1.53	
15	Neryl acetate	16.15	1485	C_12_H_20_O_2_			0.41	6.61
16	α-Longipinene	16.19	1489	C_15_H_24_				3.18
17	α-Ylangene	16.59	1519	C_15_H_24_				0.11
18	δ-Elemene	16.68	1525	C_15_H_24_	1.00	0.31	0.22	
19	Modephene	16.75	1527	C_15_H_24_				8.49
20	β-Elemene	16.79	1533	C_15_H_24_	13.61	6.03	2.77	
21	α-Isocomene	16.85	1538	C_15_H_24_				10.27
22	β-Isocomene	17.20	1564	C_15_H_24_				4.63
23	β-Caryophyllene	17.33	1574	C_15_H_24_	4.00	5.27	5.02	1.66
24	γ-Curcumene	17.40	1579	C_15_H_24_				1.59
25	β-Farnesene	17.58	1593	C_15_H_24_			52.53	0.83
26	α-Caryophyllene	17.82	1611	C_15_H_24_	0.79	0.85	3.67	0.66
27	α-Bergamotol	17.94	1620	C_15_H_24_O			1.10	
28	β-Bergamotene	18.02	1625	C_15_H_24_				0.63
29	β-Sesquiphellandrene	18.02	1625	C_15_H_24_				15.91
30	Germacrene D	18.20	1639	C_15_H_24_	0.79	0.45	6.25	
31	α-Himachalene	18.22	1641	C_15_H_24_				0.81
32	β-Selinene	18.29	1646	C_15_H_24_	0.33	0.59		
33	Bicyclogermacrene	18.42	1656	C_15_H_24_	1.40	0.55	4.70	0.34
34	Alloaromadendrene	18.55	1666	C_15_H_24_			0.09	0.81
35	β-Copaene	18.63	1671	C_15_H_24_		0.08	0.20	0.65
36	δ-Cadinene	18.71	1678	C_15_H_24_		0.11	0.33	
37	γ-Bisabolene	18.76	1682	C_15_H_24_			0.25	
38	β-Caryophyllene oxide	19.07	1704	C_15_H_24_O			0.32	
39	Nerolidol	19.13	1709	C_15_H_26_O	0.71	1.82	0.84	
40	Neryl-2-methylbutanoate	19.23	1717	C_15_H_26_O_2_				0.37
41	Farnesol	19.30	1722	C_15_H_26_O				0.30
42	Germacrene D-4-ol	19.52	1739	C_15_H_26_O	0.63	0.31	1.35	
43	Spathulenol	19.57	1742	C_15_H_24_O		0.25	0.82	
44	α-Caryophyllene oxide	19.69	1751	C_15_H_24_O		0.20	0.27	
45	6-Methyl-6-(5-methylfuran-2-yl)heptan-2-one	20.20	1790	C_13_H_20_O_2_				0.69
46	β-Atlantone	20.82	1836	C_15_H_22_O			0.54	4.86
47	Curcuphenol	21.18	1863	C_15_H_22_O				0.82
48	Zingiberenol	21.50	1887	C_15_H_26_O				1.51
49	Himachalol	21.65	1898	C_15_H_26_O			1.67	21.00
50	Aristolenol	21.68	1901	C_15_H_24_O		0.20		0.33
51	Cedrenol	21.95	1921	C_15_H_24_O		0.25		1.50
52	Neophytadiene	22.60	1970	C_20_H_38_	33.00	0.35	1.08	
53	3,7,11,15-Tetramethyl-2-hexadecene	22.68	1976	C_20_H_40_	1.23	0.20		
54	3,7,11,15-Tetramethyl-2-hexadecenol	22.89	1992	C_20_H_40_O	3.91	0.10	0.16	
55	2-Methyl-7-octadecyne	23.11	2008	C_19_H_36_	7.68	0.25	0.29	
56	2,5,5,8a-Tetramethyl-4-methylene-6,7,8,8a-tetrahydro-4H,5H-chromen-4a-yl hydroperoxide	23.36	2027	C_14_H_22_O_3_				0.57
57	Ethyl 9,12-hexadecadienoate	23.46	2035	C_18_H_32_O_2_				1.48
58	Sclareol	25.82	2212	C_20_H_36_O_2_				2.60
59	2-cis-9-Octadecenyloxyethanol	28.60	2421	C_20_H_40_O_2_		1.24		0.40
60	Methyl 5,9-docosadienoate	28.73	2430	C_23_H_42_O_2_				0.44
61	4,8,13-Duvatriene-1,3-diol	29.19	2465	C_20_H_34_O_2_	1.91	4.08	0.41	
62	8(20),14-Labdadiene-6α,13-diol	29.58	2494	C_20_H_34_O_2_		1.02		
63	8(17), 12-Labdadiene-15,16-dial	29.67	2501	C_20_H_30_O_2_		9.23		
64	Copaiferic acid	30.64	2574	C_20_H_32_O_2_	9.82	18.00	1.61	
65	Copalic acid	30.70	2579	C_20_H_32_O_2_	10.50	29.60	1.64	
66	5α-Pregnane-18,20-diol	31.10	2608	C_21_H_36_O_2_	1.76	3.31		
67	2-Methylenecholestan-3-ol	31.21	2617	C_28_H_48_O		5.30		
68	Heptacosane	31.45	2635	C_27_H_56_		1.18		
69	Isopropyl hexacosyl ether	33.24	2769	C_29_H_60_O		1.02		

L, leaves; F, flowers; S, stems; R, roots; ^a^ Compounds listed in order of elution in an HP-5MS Ultra Inert (UI) column. ^b^ Retention time in minutes. ^c^ Retention indices calculated against C_7_-C_30_ n-alkanes on the HP-5MS column. ^d^ Average relative abundance in percentage, calculated from three independent readings.

## Supplementary Materials

Supplementary materials can be found at https://www.mdpi.com/1422-0067/21/22/8566/s1. Figure S1: Variation in quality and quantity among VOCs identified from Stevia tissues—leaves, flowers, stems, and roots, Figure S2: Molecular phylogenetic analysis of SrTPSs, Figure S3: Amino acid sequence alignment of SrTPSs, Figure S4: The mass spectra of terpenoids described in Figure 4 and Figure 5, Figure S5: In vitro enzyme assay using heat-inactivated recombinant proteins, Figure S6: Expression analysis of homologs of Arabidopsis stress-induced marker genes in Stevia leaves and roots, Table S1: List of primers used in this study, Table S2: Abbreviations and accession numbers of proteins used in the phylogenetic analysis.

## Accession Numbers

The sequences of *SrTPS1-SrTPS5* were submitted to the GenBank (https://www.ncbi.nlm.nih.gov) under accession numbers MW187850-MW187854.

## Abbreviations

CaMV	Cauliflower mosaic virus
GC-MS	Gas chromatography–mass spectrometry
GPP	Geranyl pyrophosphate
GGPP	Geranylgeranyl pyrophosphate
FPP	Farnesyl pyrophosphate
ORF	Open reading frame
SG	Steviol glycoside
TPS	Terpene synthase
VOC	Volatile organic compound
YFP	Yellow fluorescent protein
