# Supplementary material for: Identification and Functional Characterization of Tissue-Specific Terpene Synthases in Stevia rebaudiana
Source: Int J Mol Sci. 2020 Nov 13;21(22):8566. doi: 10.3390/ijms21228566 (PMC7696289; doi:10.3390/ijms21228566)
Supplement: Supplementary file 1 [file ijms-21-08566-s001.zip › Supplementary Figures S1-S6.pdf]

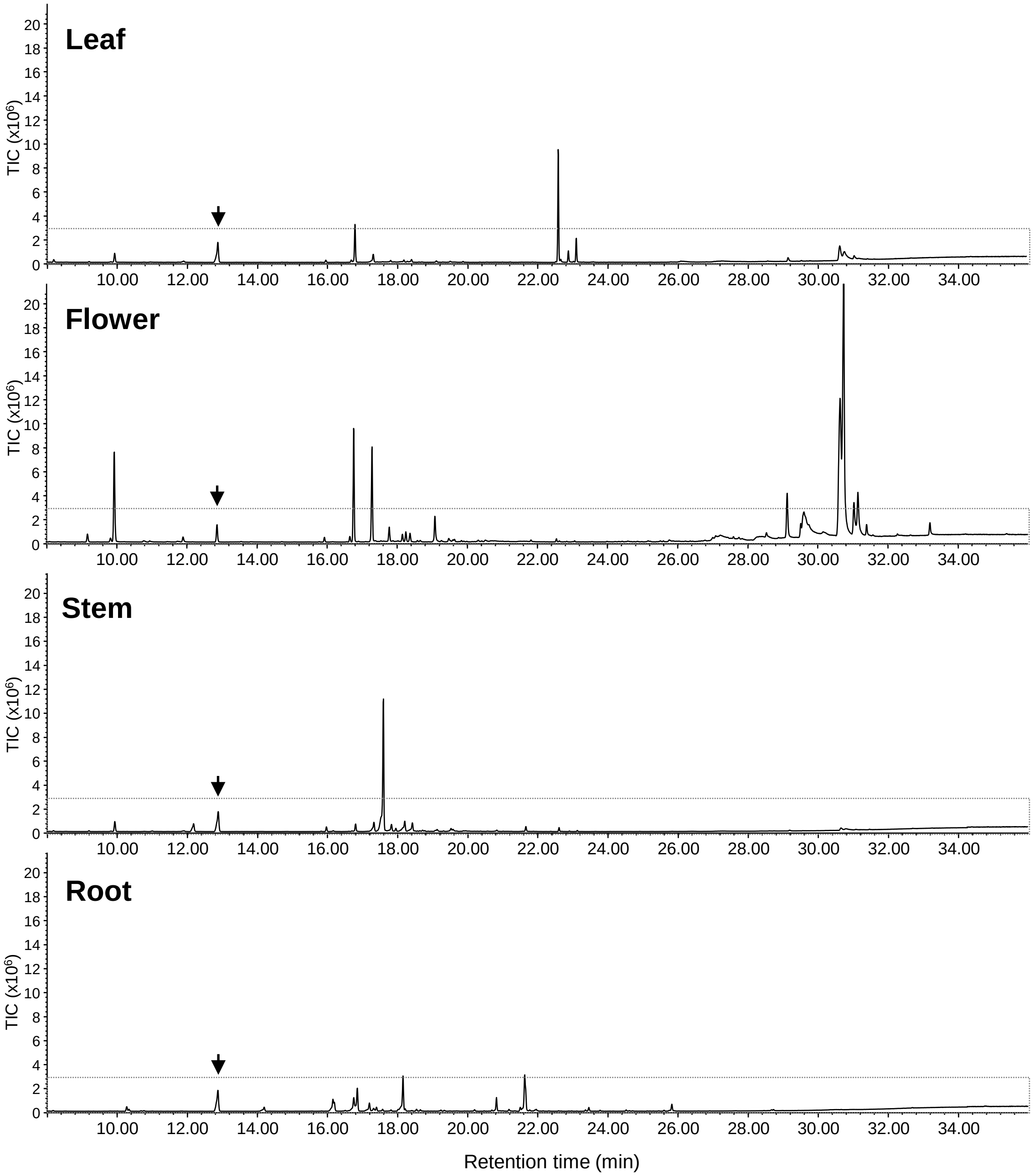

Supplementary Figure S1. Variation in quality and quantity among VOCs identified from Stevia tissues - leaves, flowers, stems and roots. The dotted regions are enlarged in Figure 1A. The arrows indicate the peak of internal standard camphor. TIC, Total ion chromatogram.

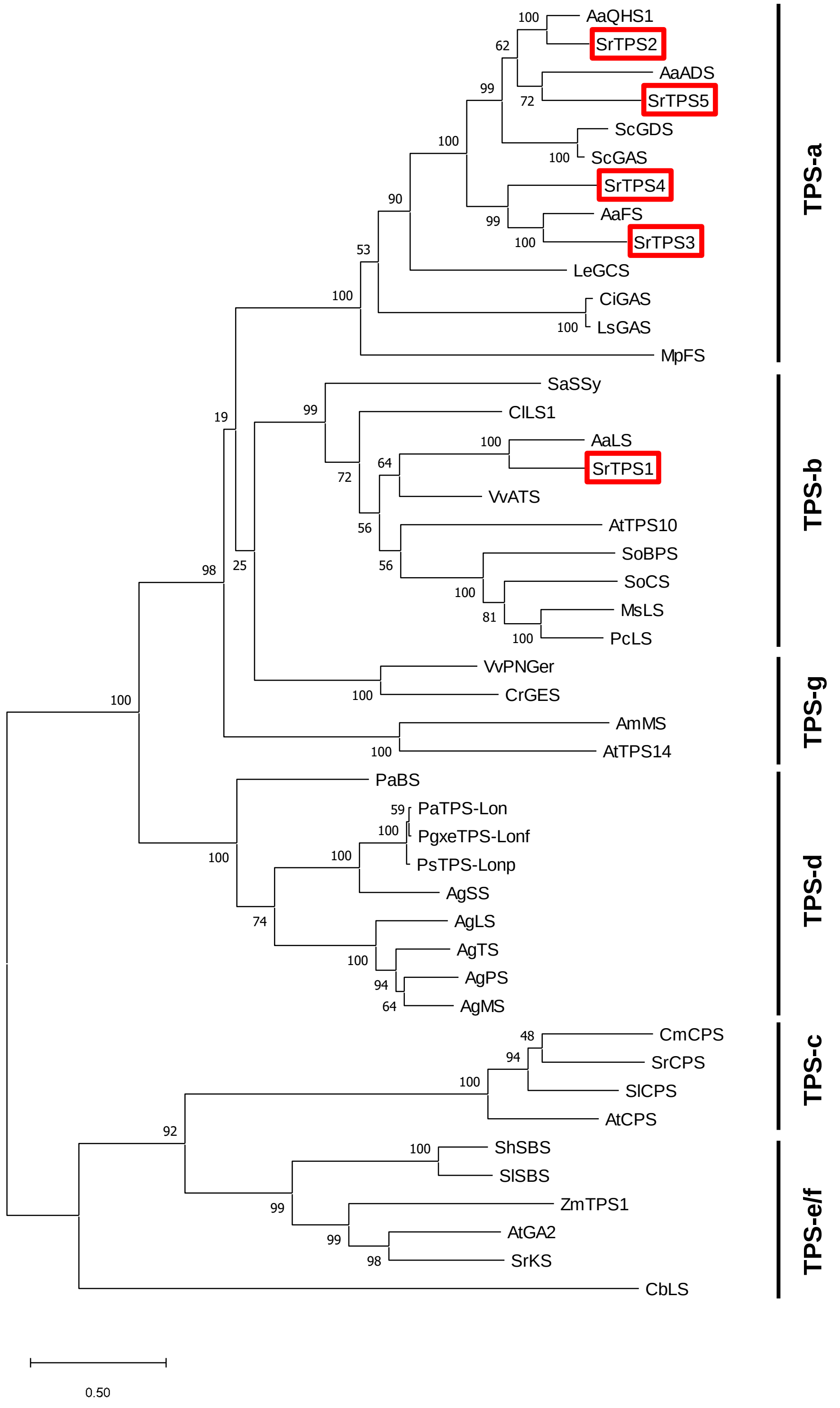

Supplementary Figure S2. Molecular phylogenetic analysis of SrTPSs. Multiple sequence alignment was carried out using CLUSTALW and the evolutionary history was inferred by using the maximum likelihood method in MEGA X. Accession numbers of proteins used in this phylogenetic analysis are provided in Table S2.

```
                                                                          R(R)X8W
SrTPS1     1 MASIFFPCSLLFCKKTHVVNSPFNVCKPVNSSQPKSIVVEPLVRRRSANYQSSVWSYEHVQSLSIKYSGKDYAARADRLK
SrTPS2     1 -----------------------------------MSIKQEEVIRPNANFPPSVWGDEFLIYEEQTGEQGDLQQRVEELK
SrTPS5     1 ------------------------------------MTSKQEAFRPFRSISPSIWGDMFLNYEKK-AELGEEDKIVENLK
SrTPS3     1 MGEL--PIN-------QFLNIAI-MSTLPISSAPFSSSSLPPVARNTVTSHPSIWGDQFLTYDEK-EDFVIEKQLVEELK
SrTPS4     1 ------------------------MS--LTSLVPNKTSFKQDVIRNSVTFHRSIWGDQFLSYDER-KDQACDQQRAKELK

SrTPS1    81 EVVKTMIQKS--SVVEYPLSSLKLVDDLKRLGIAYHFKEEISDILEMLFYQTRDKWYTM-DLNLRSLGFRLLRQHGYPVP
SrTPS2    46 ENVRKDIVSS-LDVQVEHANLLKLIDAIQRLGIAYLFEEEIKQALQHIYDTYGHEWKGE----NPSLWFRILRQQGFYVS
SrTPS5    44 EEVKKKITVA-LKSPNEHTNLLKLIDAIQRLGISYYFEEEIANALKQIYEAYGDEWNGG----SPSIWFRILRQHGFYVS
SrTPS3    70 GEVRKMLMLK-PFEPMQYVNMVKVIDVIQRLGLSYHFDGEIEDVLQHIYTTYGNNWIDEKNLESVSLWFRLLRQQGFNVS
SrTPS4    54 EKVRKELVIEGSKDPTQHIKLIELIDVVQRLGVAYHFEDEIEECLTRIYVMYGDKWINEDSLQSTSLWFRLLRQQGFKVS

SrTPS1   158 QDIFGNFKERIENLNPCQHEDMIAILNLYEASYHSFEDERILDEARDLTKKYLKENLDGIN----GSIASLVSHALEVPL
SrTPS2   121 SGIFNEYKDENGSFKESLTRDVEGLLELYEATYLGVQGEFVLEDALVFTRTHLSKIAKDLSESN-SILSTHIQDALKQPI
SrTPS5   119 CDIFNKYKDEHGAFKESLTNNVDEMLELYDATCLRVRGEVVLDEALEFTKTHLDDIAKDPHRTS-STLSAHIQDALVTPL
SrTPS3   149 SETFKNHTEENGNFKETLCNDVQGMLALYEAAYMRVEGEQVLDEALEFTKTHLAIIAKDPSCD--SLSRTEIQRALNLPL
SrTPS4   134 NEIFYKYKNEDGNFLESMKDDIHGLLSLYEATHMRVEGEDILDEALEFTKSCLTNKISNHVCINDASLETQIHQALQQPL

SrTPS1   234 HWRVPRVEARWFIEVYEKRSDANPTLIELAKLDFNMVQAIHVKDLKHASRWWRNISWDEKLTFARDRLVECFLWTVGYSY
SrTPS2   200 RKRLPRLEALRYIPFYQQQDSHNESLVELAKLGFNLLQSLHRKELSQISRWWKRLDVPNNLSYARDRMVESYFWALGVYF
SrTPS5   198 HKRIPRIEALSYIRFYEKQVSHNKSLLKLAKLGFNLLQSQHKSELNQVSKWWKDIDVQKNMPYARDRIVECYFWAFGIYS
SrTPS3   227 RRTLPRLGAVGYIPIYQQQPTHNEVLLKLAKLDFNILQSMHKKELSQLCKWWKDLDLGNRLPYVRDRCVEGYFWILAIYF
SrTPS4   214 RKRLPRLEALRYIPIYQQQESHNHDLLTFAKLDFNLLQELHRKELSQISKWWKNLDVSNKLPYVRDRIVEGYFWILAVYY
                                 DDXXD
SrTPS1   314 VPRFSLGRKDITKVNAMITTIDDVFDVYGTLDELEQFTDVISRWDINEVEELPDYMKICFLGLYNTINEIAYNTLINTRI
SrTPS2   280 EPKYSHARVFLAKIISMAAVLDDTYDVYGTYEELKIFTEAIQRWSTTCIDVLPEYMKPIYQTLLDMYKEMEEILGKEGKA
SrTPS5   278 EPKYSLARVFLAKIIQMATVVDDTFDAYGTYEELQIFTEAVERWCITCLDGLPEYMKVVYKILLDLYGEMEPIMEMQEIT
SrTPS3   307 EPEHYRTRMFLLKTCMWLVVLDDTVDNYATYEEFEIFTQAVERWSISCLDMLPEYMKLIYQQLLNHHDEMEESLEKEGKA
SrTPS4   294 EPQHSESRIFLMKACNLVIILDDTYDNYGTYEELEIFTKAVQTWSISCLDTLPEYMKPIYQELLDVHKEAEDLLEKKGKS

SrTPS1   394 LVLPYLKKAWADLCKSYLQEARWYHNGHTPTLTDYLNNACITIGTPLTFMHIMISTSINSTQEIQQWHEETENIVHYTAL
SrTPS2   360 HHLSYAKESMKEFIRSYMMEAKWANEGYVPATDEHMAVSFVSSGYGMLTTTCLVGMGDMVTDESFKWALTNPPIVKASCL
SrTPS5   358 PLFNCSKEFMIEAVKAYMVEANWVNEGHIPTIDEHTTIAFATGGGDLLISSCYLGMGDIITNNAINWAITNPPLFKASSV
SrTPS3   387 YQIHYVKEAAKDFVRSGLLEAKWLKEGYKPTLDEYISNASITIGQHVIIARSYVGRGDMVTEETFRWLATYPPLVKSSSL
SrTPS4   374 YRSYYTKEMVKEYTRNLLTEAKWVNEKYIPTVEEHMAVTLVTCAYPMIVAKCYVHGDDSVTEDTYKWLSTYPPIVKASSL
                 NSE/DTE
SrTPS1   474 IVRLADDLATSSDEMERGDIPKSIQCYMHENDATEDEARRYIKDLIMNTWKKLNEERANAKSTFQQEFIDYAMDIVRMAQ
SrTPS2   440 IARFMDDIFSQKEEKERNHVVSSVESYKKQYNVTEDYVHNVFNKKIEDAWKDLTRESLTCNKV-PMPLVIRVINLARVMD
SrTPS5   438 IGRLLNDIVSYKKEQERVHFPSFVQCYMKNHDVSEEYAINMVREEIEDVWKDINQDVLMCKDI-PRPLIVVVINYARTLY
SrTPS3   467 IFRLMDDLVTHQREQERDHIPSCIECYIKETGASKEEACVFFAKQVEDAWKVINRASLRPTDI-PFSLLSPIINIS-VIH
SrTPS4   454 ILRLMDDIATHEEEQERNHVASSIECYMKQYGVTEEHTHEVFSKLVEDNWKIINQESLRPTDV-PIPLLIPPINLARVCD

SrTPS1   554 FMYSKGDGHGHPNVT-KSHVLSLLFNPIQGI
SrTPS2   519 VLYKRKDNFTHVGEELIGHIKSLIIHDMNI-
SrTPS5   517 YLYKHNDNFSEVGEDIKDHIKSLFIHAMSI-
SrTPS3   545 VFYGGNDNYGHAGEEMIGYIKSLLVHPMVV-
SrTPS4   533 VLYTRGDYYNHAGKEMIDNIKALLVNPLSV-
```

Supplementary Figure S3. Amino acid sequence alignment of SrTPSs. Deduced amino acid sequences of SrTPSs were aligned using CLUSTALW. The transit peptide sequence of SrTPS1 is underlined in red. The domains essential for TPS activity such as R(R)X_8W motif, the Asp-rich metal cofactor binding regions, DDXXD and NSE/DTE are highlighted. Conserved amino acids are based on 100% (black), 60% (dark gray) and 30% (light gray).

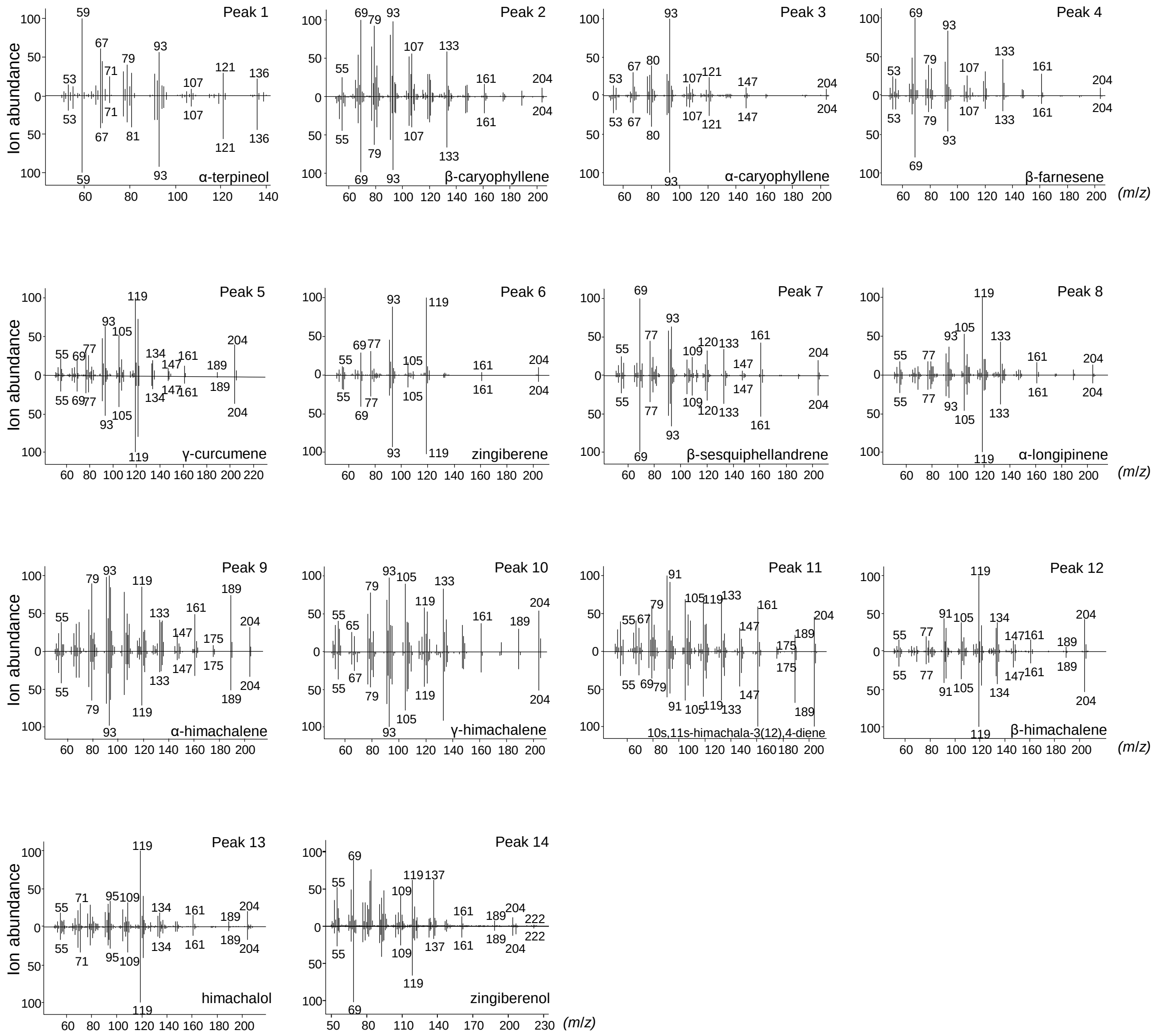

Supplementary Figure S4. The mass spectra of terpenoids described in Figures 4 and 5.

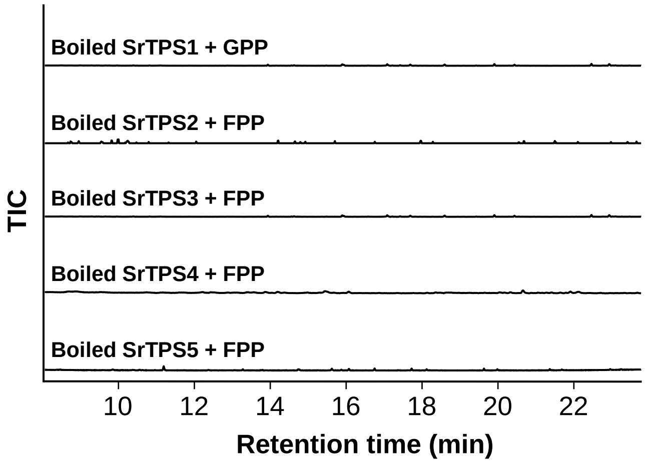

Supplementary Figure S5. *In vitro* enzyme assay using heat-inactivated recombinant proteins. TIC, total ion chromatogram.

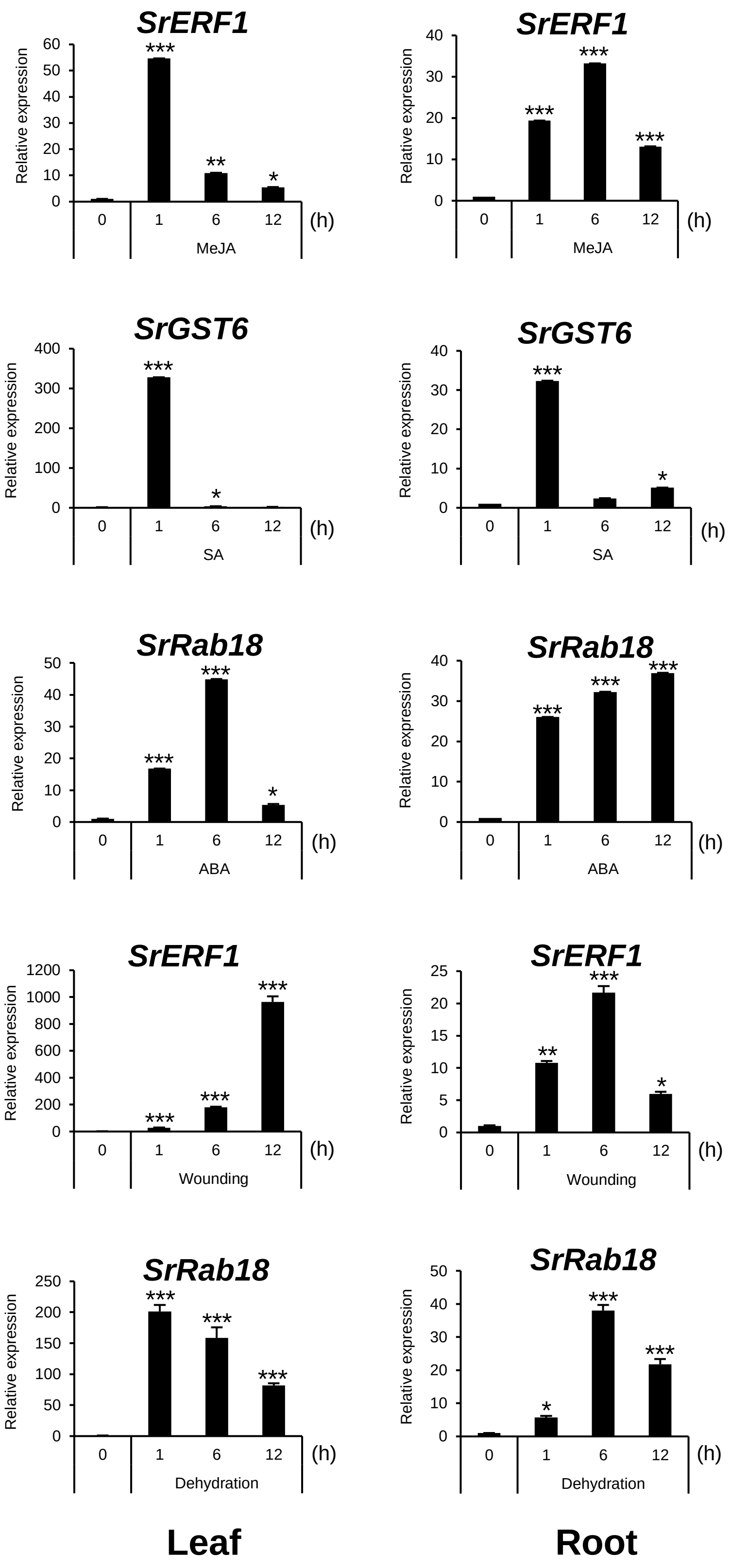

Supplementary Figure S6. Expression analysis of homologues of Arabidopsis stress-induced marker genes in Stevia leaves and roots. The housekeeping gene *actin* was used for normalization. Data is the mean ± standard deviation of three readings. Statistical significance of the measurements was determined by Student's *t*-test (* $P < 0.05$, ** $P < 0.01$, *** $P < 0.005$). MeJA, methyl jasmonate; SA, salicylic acid; ABA, abscisic acid.
